# Sclerosing mesenteritis: a case series detailing importance of early diagnosis

**DOI:** 10.1093/jscr/rjaf415

**Published:** 2025-07-11

**Authors:** Jaclyn Campbell, Nasrin Ghalyaie, Jamii St. Julien

**Affiliations:** HCA Healthcare/USF Morsani College of Medicine Bayonet Point, 14000 Fivay Rd., Hudson, FL 34667, United States; HCA Healthcare/USF Morsani College of Medicine Bayonet Point, 14000 Fivay Rd., Hudson, FL 34667, United States; HCA Florida Trinity Hospital, 9330 State Rd 54, Trinity, FL 34655, United States

**Keywords:** sclerosing mesenteritis, bowel obstruction, neuroendocrine tumor

## Abstract

Two patient cases are presented in this case series. Both patients presented with vague abdominal pain, and CT imaging showed calcified mesenteric masses. Definitive diagnoses of sclerosing mesenteritis was made on histological evaluation. Interestingly, one of these patients was initially misdiagnosed with a neuroendocrine tumor after Octreotide scan showed increased mesenteric uptake. He underwent treatment for neuroendocrine tumor however had disease progression to small bowel obstruction, requiring emergent exploratory laparotomy with palliative resection of the mesenteric mass and involved small bowel. He experienced cardiac arrest in the immediate post-operative period and unfortunately did not survive. This case series exemplifies the importance of early diagnosis and management to prevent catastrophic complications in an otherwise benign disease.

## Introduction

Sclerosing mesenteritis is an exceedingly rare disease process resulting in chronic, progressive inflammatory changes to the mesentery [1]. The etiology is unknown; however, it is often felt to be a form of autoimmune disease, paraneoplastic syndrome, lymphoma, or an altered inflammatory response to mesenteric trauma or infection [2]. Patients generally present with vague symptoms such as abdominal pain and diarrhea, but may also present with complications of bowel obstruction, obstructive uropathy, or mesenteric ischemia [1–6]. Work-up often includes biochemical analysis, CT imaging, and an Octreotide or Ga-DOTATATE scan to rule out neuroendocrine tumors. However, false-positive results can occur in patients with inflammatory disease processes [7–10]. Due to these non-specific presenting symptoms, misdiagnosis and delay of treatment is common. Definitive diagnosis is made on histological evaluation. Treatment includes outpatient surveillance in asymptomatic patients, corticosteroids, antiestrogens such as tamoxifen, and escalation to immunotherapy as the disease progresses [11, 12].

## Case 1

**Figure 1:**
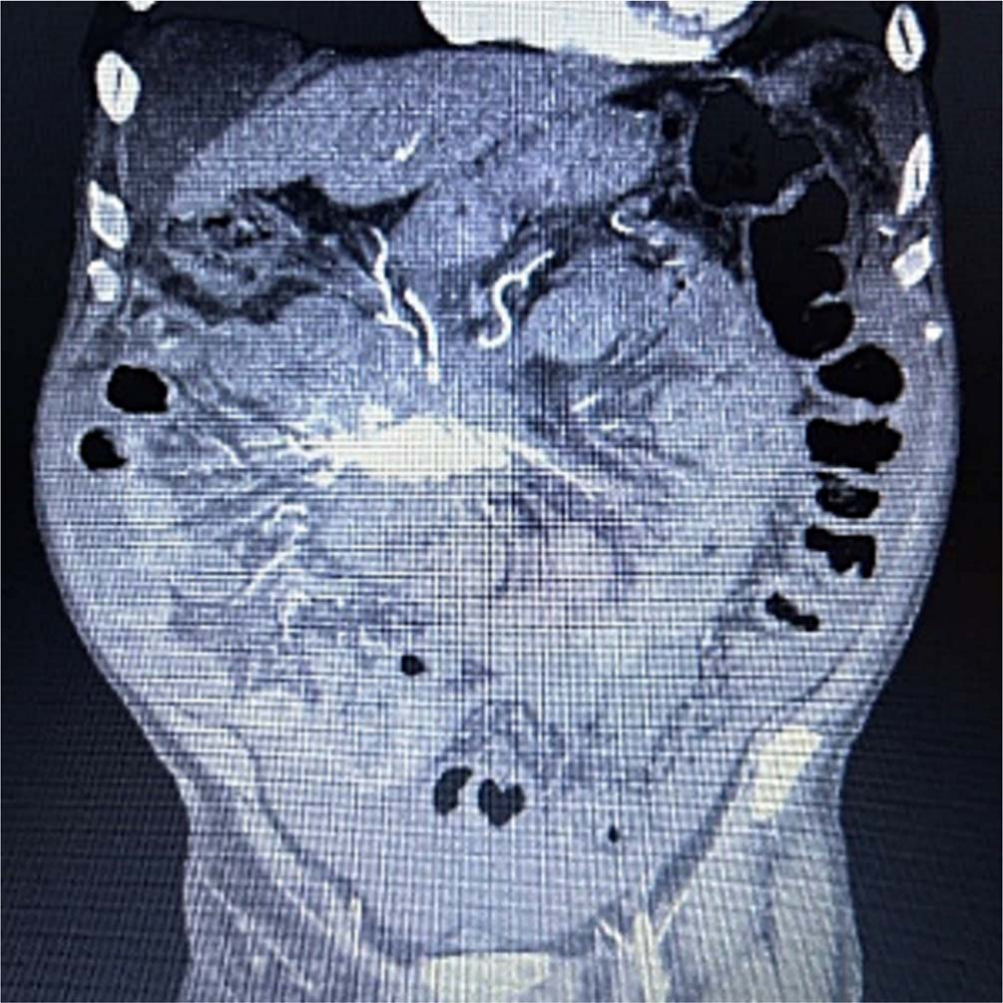
Anterior–posterior view of CT abdomen/pelvis scan showing large, central calcified mesenteric mass, Case 1.

**Figure 2:**
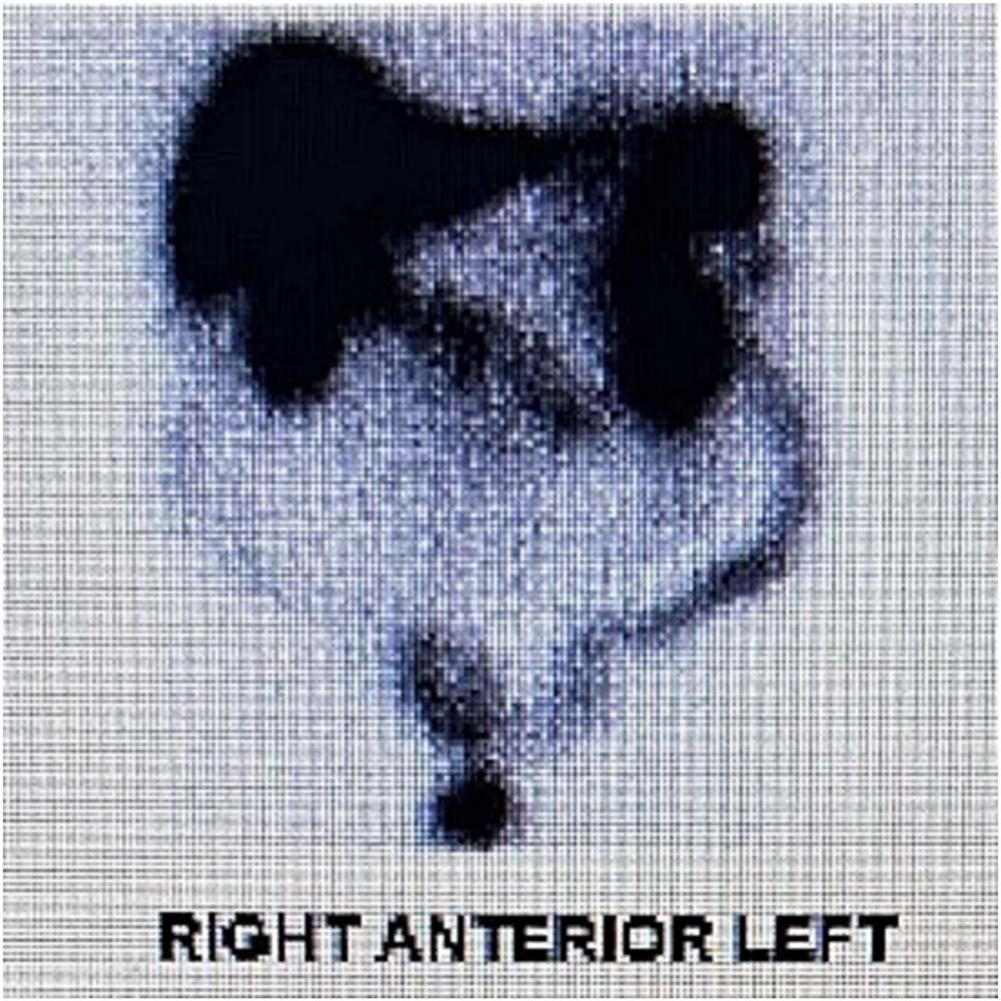
Gallium DOTATATE scan showing increased uptake at the location of the mesenteric mass, Case 1.

**Figure 3:**
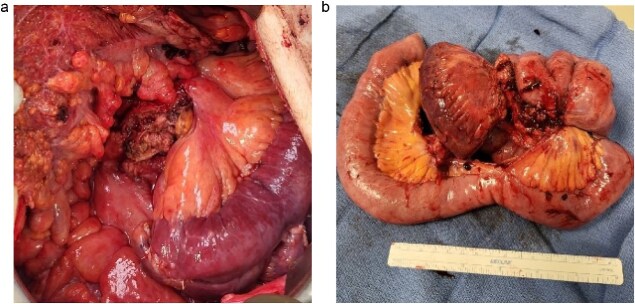
Small bowel and mesenteric mass resection specimens, Case 1.

A 83-year-old male presented to his primary care physician with a 6-week history of vague postprandial abdominal pain with associated diarrhea and a 5-lb weight loss. CT abdomen/pelvis showed a 7 × 5 cm spiculated mass at the root of the mesentery involving the superior mesenteric vein and multiple mesenteric arterial branches, and associated small bowel edema and thickening (Fig. 1). He was referred to a surgical oncologist. Tumors markers including CEA, CA 19–9, Chromogranin A, serum serotonin, and urinary 5-HIAA were unremarkable. Octreotide scan showed mild central uptake at the location of the mesenteric mass (Fig. 2). Diagnostic laparoscopy showed mesenteric mass at the root of the mesentery and biopsy revealed fibrous tissue with calcifications and no evidence of malignancy. He was referred to a medical oncologist and began Lanreotide injections for presumed neuroendocrine tumor. He completed 12-months of Lanreotide therapy, however he remained symptomatic with abdominal pain, diarrhea, and ongoing weight loss. He was referred to a tertiary cancer center, but no treatment was offered in the absence of proven malignancy. He then saw another surgeon who obtained a Ga-DOTATATE PET CT scan which showed moderate ascites, an increase in size of the mesenteric mass to 7.3 × 6.3 cm, low level of tracer at the periphery of the mass, and intense activity within the 2^nd^ and 3^rd^ portions of the duodenum. He was referred to an advanced endoscopist for EGD/EUS, which did not show a duodenal mass. Due to ongoing symptoms he was scheduled for repeat diagnostic laparoscopy with possible resection of mesenteric mass versus repeat biopsy. On the morning of surgery, he presented with dizziness and acutely worsening abdominal pain. He was tachycardic and hypotensive with abdominal distention and peritonitis on exam. Due to concern for a perforated viscus the decision was made to proceed with exploratory laparotomy. He was found to have purulent ascites and dusky small bowel with edema and central swirling atop a large, calcified mesenteric mass. Upon further inspection, a single site of small bowel perforation was identified and there was leakage of succus into the abdominal cavity. A palliative resection of the perforated small bowel and adjacent mesenteric mass was performed. 100 cm of small bowel was resected en bloc with the mesenteric mass, but residual gross tumor was left at the base of the superior mesenteric artery and encasing the middle colic artery (Fig. 3). Intra-operative doppler and Firefly were used to determine perfusion of the remaining 120 cm of small bowel and a side-to-side small bowel anastomosis was performed. He was hemodynamically stable at the termination of the procedure, extubated, and admitted to the intensive care unit for close observation. Post-operatively, he converted into atrial fibrillation with rapid ventricular response and ultimately died from cardiac arrest secondary to a massive myocardial infarction. Pathology was significant for sclerosing mesenteritis without evidence of malignancy.

## Case 2

**Figure 4:**
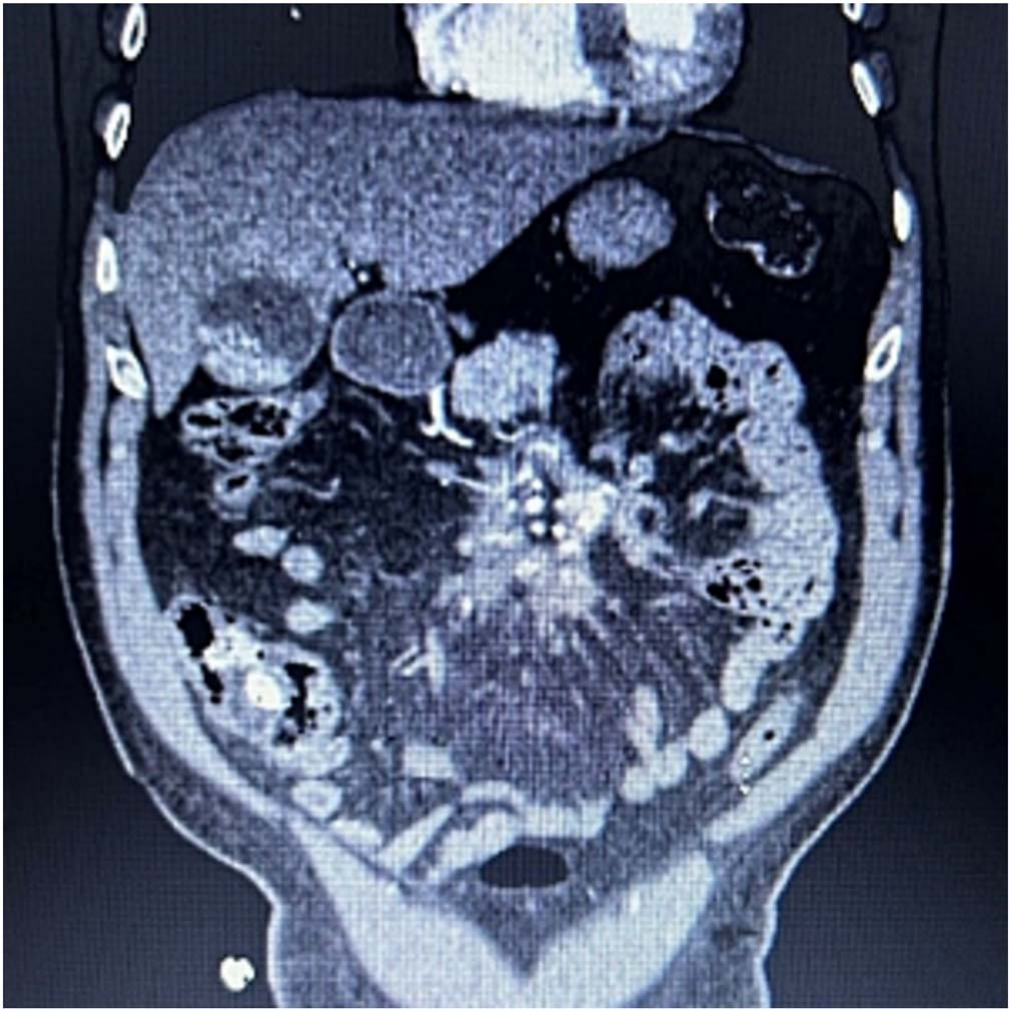
CTA abdomen/pelvis showing central calcified mesenteric mass, Case 2.

A 76-year-old male who presented to the outpatient clinic for evaluation of a central mesenteric mass. Patient reported a several year history of vague abdominal pain which progressively worsened over the few months prior to presentation. He denied any history of abdominal trauma or associated symptoms such as weight loss, flushing, or diarrhea. 24-h urine studies and serum serotonin levels were unremarkable. CTA of the abdomen and pelvis showed a 2.5 × 1.5 × 2.7 cm calcified mesenteric mass in the mesenteric root with tethering to the surrounding the mesenteric vessels and no evidence of bowel obstruction (Fig. 4). Upper and lower endoscopy were performed and were unremarkable. A Ga-DOTATATE PET can showed an infiltrating soft tissue density with calcification in the mesenteric root measuring 2.4 × 4.4 cm, but without prominent uptake (maximum SUV of 2). No other lesions were noted. Patient subsequently underwent elective exploratory laparotomy with incisional biopsy of central mesenteric mass. Intraoperatively, an infiltrative, calcified soft tissue mass within the central mesentery with encasement and tethering of the superior mesenteric artery was found. There was no evidence of bowel perforation or obstruction. Preliminary pathology was consistent with sclerosing mesenteritis. Specimen was sent for further analysis at Cleveland Clinic’s Robert J. Tomsich Pathology and Laboratory Medicine Institute (RT-PLMI). Further analysis did not show evidence of neoplasm and all neuroendocrine markers were negative, confirming the diagnosis of sclerosing mesenteritis. Patient continued to report vague abdominal discomfort post-operatively and was referred to rheumatology for consideration of anti-inflammatory therapy and tamoxifen.

## Discussion

Sclerosing mesenteritis is an exceedingly rare disease affecting ˂1% of the population [13]. It is a term often used to broadly describe mesenteric inflammatory processes; however, a more accurate description is that of a spectrum of disease in which there is gradual progression from acute mesenteric fat necrosis (mesenteric lipodystrophy) to chronic mesenteric inflammation (mesenteric panniculitis), and finally to mesenteric fibrosis (sclerosing mesenteritis) [1]. Although the etiology and pathogenesis of sclerosing mesenteritis are unclear, there are several reports that depict an association with autoimmune disorders, Non-Hodgkin lymphoma and other paraneoplastic syndromes, and an abnormal genetic response to mesenteric trauma and/or infection [2]. Roughly 10%–15% of mesenteric lesions are found incidentally in asymptomatic patients. However, patients can present with a range of symptoms including isolated vague abdominal pain in up to 70%, systemic manifestations of fever, generalized malaise, and weight loss (23%), and inconsistent bowel habits with either constipation or diarrhea (20%) [4]. Additionally, patients may present with complications such as bowel obstruction in up to 24%, obstructive uropathy (24%), chylous ascites (14%), and chronic mesenteric ischemia [1, 2, 4].

Clinical work-up includes biochemical testing and specialized imaging to rule out associated disease processes such as gastroenteropancreatic neuroendocrine tumors, autoimmune disorders, Non-Hodgkin lymphoma, and other paraneoplastic syndromes. Definitive diagnosis is provided by tissue biopsy for histological evaluation, which confirms mesenteric fibrosis and simultaneously rules out other pathologies. Treatment is determined based on patient presentation. Asymptomatic patients typically do not require treatment and can undergo outpatient surveillance [2, 4]. Symptomatic patients without evidence of obstructive complications are initiated on 3-month course of tamoxifen and prednisone [2, 4]. Tamoxifen is a selective estrogen receptor modulator with the ability to inhibit fibroblast production of transforming growth factor-beta one (TGF-β1), and is ultimately thought to slow the progression of mesenteric fibrosis [12]. If symptoms improve, prednisone is tapered and tamoxifen is continued indefinitely [4]. If symptoms persist or progress, medical management is escalated to immunomodulatory therapy with azathioprine, followed by cytotoxic therapy with cyclophosphamide if symptoms persist or progress, and finally thalidomide for refractory disease [4].

For both cases presented here, surgery was required to establish the diagnosis. The patient in Case 1 was treated for presumed neuroendocrine tumor based on increased uptake noted on Ga-DOTATATE PET scan, and an initially non-diagnostic biopsy. He continued to have worsening symptoms and ultimately developed obstructive complications requiring emergent surgery, after which he was diagnosed with sclerosing mesenteritis. There have been numerous case reports of Ga-DOTATATE PET scans yielding false-positive results for neuroendocrine tumors in patients with inflammatory processess [7–10]. These false-positive results can complicate the diagnosis, delay initiation of appropriate treatment, and ultimately allow progression of the disease. In the literature, sclerosing mesenteritis is labeled as a benign, progressive inflammatory process. Case 1 demonstrates how this disease can quickly become life-threatening, and underscores the importance of early diagnosis for proper disease management.

## Conclusion

Sclerosing mesenteritis is a rare disease process that falls on the severe end of a spectrum of chronic inflammatory changes to the mesentery. The etiology is unknown and diagnosis can be challenging due to non-specific symptoms at presentation and poor specificity of imaging studies. Definitive diagnosis is confirmed on histological evaluation, which should be obtained quickly in the symptomatic patient. Treatment consists primarily of medical therapies. Corticosteroids and tamoxifen are utilized to prevent disease progression, followed by escalation to immunotherapy and cytotoxic agents as the disease progresses. Overall, early diagnosis is essential for proper management.
